# OrthoFiller: utilising data from multiple species to improve the completeness of genome annotations

**DOI:** 10.1186/s12864-017-3771-x

**Published:** 2017-05-18

**Authors:** Michael P. Dunne, Steven Kelly

**Affiliations:** Department of Plant Sciences, University of Oxford, South Parks Road, Oxford, OX1 3RB UK

**Keywords:** Genome annotation, Gene prediction, Orthology, Orthogroup

## Abstract

**Backround:**

Complete and accurate annotation of sequenced genomes is of paramount importance to their utility and analysis. Differences in gene prediction pipelines mean that genome annotations for a species can differ considerably in the quality and quantity of their predicted genes. Furthermore, genes that are present in genome sequences sometimes fail to be detected by computational gene prediction methods. Erroneously unannotated genes can lead to oversights and inaccurate assertions in biological investigations, especially for smaller-scale genome projects, which rely heavily on computational prediction.

**Results:**

Here we present OrthoFiller, a tool designed to address the problem of finding and adding such missing genes to genome annotations. OrthoFiller leverages information from multiple related species to identify those genes whose existence can be verified through comparison with known gene families, but which have not been predicted. By simulating missing gene annotations in real sequence datasets from both plants and fungi we demonstrate the accuracy and utility of OrthoFiller for finding missing genes and improving genome annotations. Furthermore, we show that applying OrthoFiller to existing “complete” genome annotations can identify and correct substantial numbers of erroneously missing genes in these two sets of species.

**Conclusions:**

We show that significant improvements in the completeness of genome annotations can be made by leveraging information from multiple species.

**Electronic supplementary material:**

The online version of this article (doi:10.1186/s12864-017-3771-x) contains supplementary material, which is available to authorized users.

## Background

Genome sequences have become fundamental to many aspects of biological research. They provide the basis for our understanding of the biological properties of organisms, and enable extrapolation and comparison of information between species. Owing to the increasing availability and affordability of whole-genome sequencing technology [1, 2], genomic data sets are now produced at a rate at which it is infeasible to rely entirely on careful manual curation to annotate a new genome; rather it is taken as given that a considerable portion of the process must be automated.

There has been substantial methodology development in the area of automated gene prediction, with the production of several effective algorithms for identifying genes in *de novo* sequenced genomes [3]. In general, these methods predict genes by learning species-specific characteristics from training sets of manually curated genes. These characteristics include the distribution of intron and exon lengths, intron GC content, exon GC content, codon bias, and motifs associated with the starts and ends of exons (splice donor and acceptor sites, poly-pyrimidine tracts and other features). These characteristics are then used to identify novel genes in raw nucleotide sequences. These prediction methods vary in their performance, as demonstrated by considerable disagreement in the genes and gene models that they predict [3, 4]. For example, one study [4] comparing Augustus, Fgenesh, GENSCAN and MAKER, looked at the number of genes predicted on a sample set of *D. melanogaster* assemblies with varying numbers of scaffolds. At the extreme end, with 707 scaffolds, the most frugal prediction (MAKER, with 12687 predicted genes) was almost doubled by the most generous prediction (GENSCAN, with 22679 predicted genes). Thus it is to be expected that genome annotations generated by different research groups using different methodologies will differ considerably in the complement of genes that they contain. This disparity is exemplified by a recent study [5] that analysed 12 published plant genomes, assessing them for completeness relative to highly conserved gene sets such as BUSCO [6] and CEGMA [7]. The study found strong evidence for universal eukaryotic genes which appeared to be present in the genomes but had no corresponding gene annotations. This indicates that many genomes likely lack gene annotations even for highly conserved genes.

Absent or inaccurate gene models can not only contribute to oversights in biological investigations, they can also lead to false assertions in large-scale genome and cross-species analyses [8]. For example, incorrectly missing gene annotations can be mistakenly interpreted as gene loss, and such interpretations can lead to mistaken inferences about the biological or metabolic properties of an organism. Similarly, missing gene models can lead to errors in gene expression analyses that map and quantify RNA-seq reads using predicted gene models. Here, reads derived from erroneously missing genes, as they have no reference to map to, have the potential to map to the wrong gene leading to errors in transcript abundance estimation.

Much of the cost and effort involved in *de novo* genome annotation can be reduced by leveraging data from other taxa. Moreover, data from disparate taxa have the potential to be used simultaneously to improve a cohort of genome annotations in a mutualistic framework. A number of approaches have been developed to utilise data from other species to improve or assist the process of genome annotation. For example, an automated alignment-based fungal gene prediction (ABFGP) method [9] has been developed for fungal genomes. While this method works well on fungal genomes, it cannot be applied to other taxa and thus has limited general utility.

OrthoFiller aims to simultaneously leverage data from multiple species to mutually improve the genome annotations of all species under consideration, using the concept of orthogroups. It is designed specifically to find “missing” genes in sets of predicted genes from multiple species. That is, to identify those genes that should be present in a genome’s annotation, whose existence can be verified through comparison with known gene families. A standalone python implementation of the algorithm is available under the GPLv3 licence at https://github.com/mpdunne/orthofiller. Example datasets and instructions for running the algorithm are included in the git repository.

## Results

### Problem definition, algorithm overview and evaluation criteria

OrthoFiller aims to find genes that are present in a species’ genome, but which have no predicted gene model in the genome annotation for that species. It takes a probabilistic, orthology-based approach to gene identification, leveraging information from multiple species simultaneously to improve the completeness of the genome annotations for all species under consideration. OrthoFiller is not designed for *ab initio* gene prediction and requires that each genome under consideration possesses a basic level of annotation, taken to be at least 100 annotated genes. The genomes should ideally be from a set of related species from the same taxonomic group (genus, family, order or class).

OrthoFiller makes use of the concept of orthogroups, an extension of the pairwise concepts of orthologous and paralogous genes. For a given set of species, an orthogroup is the set of genes descended from a single ancestral gene in the last common ancestor of those species [10]. By definition, orthogroups may contain paralogous as well as orthologous genes. In the case of OrthoFiller, orthogroups provide the basis for the gene searching process, the aim being specifically to find unannotated members of existing orthogroups. As a result, OrthoFiller is able to find genes that are paralogous as well as orthologous to known genes.

**Fig. 1:**
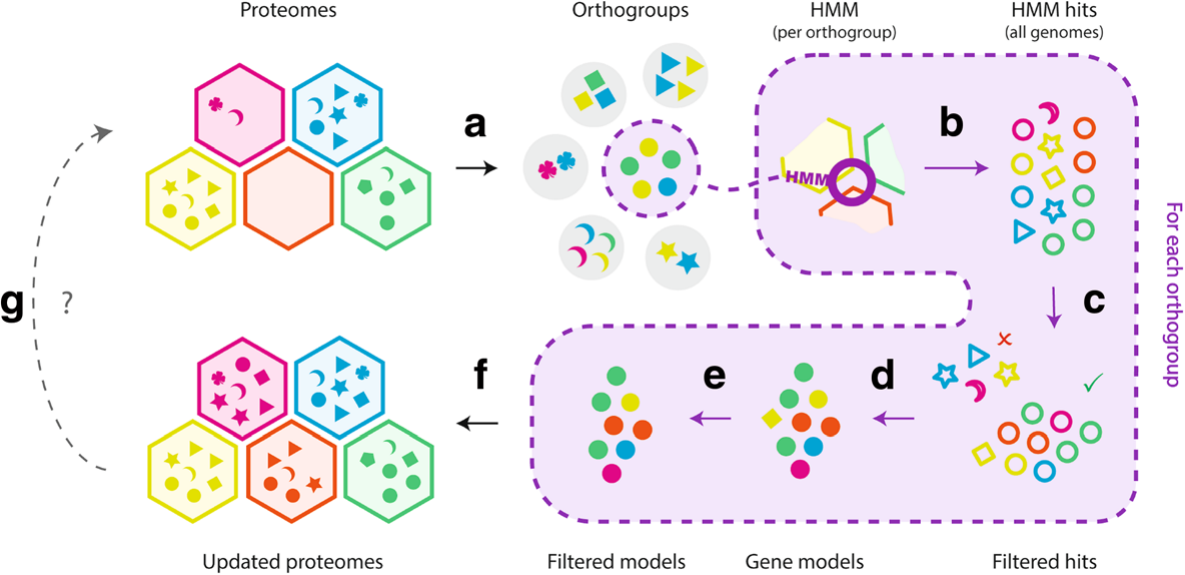
Workflow diagram for the OrthoFiller algorithm. **a** Proteomes are subdivided into orthogroups using OrthoFinder. **b** Protein sequences in each orthogroup are subject to multiple sequence alignment, back-translated to DNA and used to create hidden Markov models (HMMs). These HMMs are used to search each genome in the set. **c** The set of hits are evaluated and filtered to remove low quality hits. **d** Gene models are constructed around each retained hit using Augustus. **e** The new gene models are compared to the hints that were used to generate them, and filtered to remove those which bear insufficient similarity to the hints. **f** The filtered genes are clustered into orthogroups and genes that are successfully placed into the orthogroup that was used to identify them are retained. **g** The process may be run once, or iteratively until no further genes are found

A workflow for OrthoFiller is shown in Fig. 1. The basic input for the algorithm is a set of genome annotation files in general transfer format (GTF) and a set of corresponding genome sequence files in FASTA format. The output from the algorithm is a set of FASTA format amino acid sequences for each inputted species, and a set of GTF annotations containing the new genes. OrthoFiller uses OrthoFinder [10] to cluster the inputted genes into orthogroups. Advanced users may also specify their own orthogroups, as described in the software documentation.

Protein sequences are extracted from the genome FASTA files using the coordinates in the GTF files and a user-selected translation table. OrthoFiller then uses OrthoFinder to cluster the genes from the species into orthogroups. The protein sequences of each orthogroup are aligned and the source nucleotide sequences for these proteins are threaded back through the protein multiple sequence alignment to create multiple sequence alignments of the nucleotide sequences of each orthogroup. Each nucleotide alignment is used to build a hidden Markov model (HMM) that is used to search the complete genome sequence of each species under consideration. The scores of these HMMs are used to learn the score distributions of true positive and false positive HMM hits (see Implementation). Each hit to an HMM that does not overlap with an existing predicted gene is subject to filtration using species-specific parameters that have been learned for true and false positive hits. Each hit that survives this filtration is considered to be a potential genic region, or *hint*. The algorithm then attempts to build gene models around these hints, using the Augustus [11] gene finder. Gene models constructed by Augustus are subject to two successive rounds of assessment and filtration. Firstly, the predicted gene models are compared against the hints that were used to inform them: if the gene model and its source hint are not sufficiently similar (see Implementation), the gene model is considered to be unrelated to the hint, and thus to the orthogroup used to inform its prediction. Secondly, the newly predicted genes that satisfy the first criterion are subject to orthogroup inference using the full set of existing and newly predicted genes. Those newly predicted genes that are clustered in an orthogroup whose HMM was used to predict them are then accepted as *bona fide* genes and added to the genome annotation. Thus, genes predicted by OrthoFiller satisfy stringent orthology-based criteria for their existence.

To demonstrate the utility of OrthoFiller on real data it was applied independently to two sets of species. Set A comprised five fungal genomes (Table 1) and Set B comprised five plant genomes (Table 2), sourced from the Joint Genome Institute (JGI) and the Saccharomyces Genome Database (SGD) [12–15]. OrthoFiller was assessed using these datasets in two ways: first via simulating an incomplete genome annotation by randomly removing entries from the genome annotation of one species from each set, and assessing the accuracy of OrthoFiller in recovering the removed genes; second by application of OrthoFiller to the complete datasets and validating the novel detected genes through analysis of publicly available RNA-seq data.

**Table 1:** Species Set A, fungal species used for algorithm validation

Species name	Source	Strain	Taxonomy ID	References
*Eremothecium gossypii*	JGI^a^	*ATCC10895*	284811	[12]
*Debaromyces hansenii*	JGI	*CBS767*	284592	[13, 14]
*Kluveromyces lactis*	JGI	*CLIB210*	284590	[13]
*Saccaromyces cerevisiae*	SGD^b^	*S288C*	559292	[26]
*Yarrowia lipolytica*	JGI	*CLIB122*	284591	[13]

^a^Joint Genome Institute; ^b^Saccaromyces Genome Database

**Table 2:** Species Set B, plant species used for algorithm validation

Species name	Source	Version	Taxonomy ID	References
*Arabidopsis thaliana*	JGI	TAIR10	3702	[15]
*Brassica rapa*	JGI	v1.3	3711	[15]
*Carica papaya*	JGI	ASGPBv0.4	3649	[15]
*Capsella rubella*	JGI	v1.0	81985	[15]
*Theobroma cacao*	JGI	v1.1	3641	[15]

Two measures were used to assess the quality of recovered genes: the protein F-score and the orthogroup F-score, both defined in the Implementation section. These scores were calculated for all genes identified by OrthoFiller, by comparing the recovered gene with the removed gene and assuming that the original removed gene model was correct. Genes that are unique to the test species that lack homologues in other species were not analysed in this test, as OrthoFiller was designed to find evolutionarily conserved genes. As there were no publicly-available comparable methods that perform the same task as OrthoFiller, the method was assessed in comparison to performing the analysis without conducting the OrthoFiller evaluation and filtration steps. i.e. using unfiltered HMM hits from the orthogroups as hints for the *de novo* gene finder Augustus, and accepting all identified gene models that did not overlap an existing gene.

### Evaluation of OrthoFiller on *S. cerevisiae* after removal of 10% of gene annotations

**Fig. 2:**
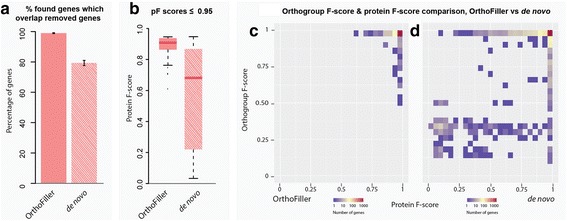
Performance of OrthoFiller on *S. cerevisiae* genome with 10% of annotated genes removed. **a** Using OrthoFiller an average of 158 of 160 found genes (99.0%) had genomic locations matching any of the 513 deleted genes. In the absence of OrthoFiller filtration an average of 447 of 582 (79.2%) of the found genes matched any of the deleted genes. **b** Boxplot of protein F-scores for genes predicted using OrthoFiller, versus predictions made in the absence of OrthoFiller filtration, that had a protein F-score of ≤0.95. **c** Density plot showing the protein and orthogroup F-scores for all recovered genes using OrthoFiller. **d** Density plot showing the protein and orthogroup F-scores for all recovered genes in the absence of OrthoFiller filtration

Figure 2 and Table 3 show the results of running OrthoFiller on the set of fungal species shown in Table 1 after random removal of 10% of “discoverable” genes (genes that were contained in an orthogroup with at least one gene from another species) from the predicted complement of genes in *S. cerevisiae* (i.e. 513 nuclear encoded gene annotations were deleted from a total set of 5129 discoverable genes). This was performed 10 times, each time with a different disjoint subset removed. The full details of detection of the deleted genes at different stages in the OrthoFiller algorithm are shown in Additional file 1: Figure S1.

**Table 3:** Recovery of removed genes in *S. cerevisiae, averaged over 10 runs*

	10% annotations removed				90% annotations removed			
	OrthoFiller		*de novo*		OrthoFiller		*de novo*	
No. genes removed	513		513		4615		4615	
Total genes found^a^	160	(11.4)	582	(21.2)	1473	(47.5)	4270	(28.2)
Found genes which overlap removed genes^a^	158	(11.2)	462	(11.9)	1472	(47.4)	4163	(18.3)
Total recovered genes^a^	158	(11.1)	447	(9.6)	1471	(47.3)	4074	(22.4)
Number of split genes^a^	0	(0.3)	11	(4.5)	1	(0.7)	89	(8.49)
Mean pF-score of found genes^a^	0.99	(<0.01)	0.95	(0.01)	0.99	(<0.01)	0.96	(<0.01)
Mean oF-score of found genes^a^	0.99	(<0.01)	0.96	(0.01)	0.99	(<0.01)	0.96	(<0.01)
High-quality found genes (pF-score ≥0.95)^a^	152	(9.1)	412.3	(9.4)	1401.7	(44.76)	3766	(33.6)
Lower-quality found genes (pF-score <0.95) ^a^	6.2	(3.2)	49.3	(12.2)	70.6	(5.6)	396.7	(23.1)
Mean pF-score of lower-quality genes^a^	0.89	(0.04)	0.57	(0.07)	0.88	(<0.01)	0.63	(0.01)
% of lower-quality genes with oF-score <0.95^a^	4.0	(8.1)	42.1	(9.3)	4.8	(1.4)	38.7	(1.1)

^a^Numbers shown are rounded mean values from 10 disjoint removed subsets of genes, with standard deviations bracketed

After running OrthoFiller, an average of 160 genes were predicted in the genome of *S. cerevisiae* that were not present in the submitted depleted genome annotation file. Of these, 98.9% overlapped with genes that were deleted from the original annotation. 96.1% of genes were recovered to high accuracy (protein F-score ≥ 0.95). The mean protein F-score of the remaining genes of lower accuracy (protein F-score < 0.95) was still high at 0.89 (Fig. 2b). All of the genes that had lower gene model accuracy were placed in exactly the same orthogroup as expected when the sequences were subjected to orthogroup inference. Thus, although typically around six of the gene models differed from the original reference gene model, this difference was not sufficient to disrupt downstream identification of orthogroups.

To provide a comparison, in the absence of the OrthoFiller evaluation steps an average of 582 genes were identified, of which only 79.2% overlapped with genes that were deleted from the original annotation and an average of 120 genes had not been predicted as genes in the original *S. cerevisiae* genome (Fig. 2a), indicating many of these were erroneous. In addition, on average 11 removed genes were split into multiple individual genes upon recovery. Of the predicted genes that overlapped with removed genes, 89.3% were recovered with high accuracy (protein F-score ≥ 0.95) and the mean protein F-score of those recovered to a lower accuracy was 0.57 (Fig. 2b), considerably lower than with OrthoFiller. Of the lower-quality genes, an average of 42.1% had an orthogroup F-score less than or equal to 0.95, compared with 4.0% for OrthoFiller. Moreover, 39.4% of the lower-quality genes were sufficiently mis-predicted that they failed to be placed in an orthogroup, or were placed in an orthogroup that shared no members with the orthogroup that contained the original gene. Thus in the absence of OrthoFiller filtration there was an increase in the percentage of gene prediction errors and a reduction in the accuracy of orthogroup inference.

Figure 2c-d show the distribution of orthogroup F-scores versus protein F-scores obtained following application of OrthoFiller to this test dataset. In these figures, results from all 10 runs have been pooled together. The majority of genes recovered with OrthoFiller had both high protein and orthogroup F-scores (Fig. 2c): 94.4% had both F-scores ≥ 0.95. This indicates that the majority of predicted genes were identical (or nearly identical) to the original removed gene and that when subjected to orthogroup inference they were clustered in the correct orthogroup. Imperfect protein F-scores can be explained by discrepancies in intron/exon and start/stop codon choices between the removed and recovered gene models. Imperfect orthogroup F-scores were due to fluctuations in orthogroup membership. Figure 2d shows the results in the absence of OrthoFiller processing. In this case, 85.3% were of dually high quality. However, 3.2% of predicted genes had both a low (<0.5) protein and orthogroup F-score, indicating those predicted genes were sufficiently incorrect to cause errors in orthogroup inference. Thus, although OrthoFiller does not recover all deleted genes (30.8% of removed genes), application of OrthoFiller resulted in the recovery of high-quality gene annotations that contain few (in this example there are none) incorrectly predicted genes.

### Evaluation of OrthoFiller on *S. cerevisiae* after removal of 90% of gene annotations

**Fig. 3:**
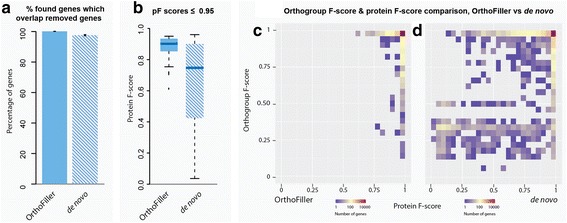
Performance of OrthoFiller on S. cerevisiae genome with 90% of annotated genes removed. **a** Using OrthoFiller an average of 1472 of 1473 found genes (>99.9%) matched any of the 4615 deleted genes. In the absence of OrthoFiller filtration an average of 4163 of 4267 (97.5%) of the found genes matched any of the deleted genes. **b** Boxplot of protein F-scores for genes predicted using OrthoFiller,  versus predictions made in the absence of OrthoFiller filtration, that had a protein F-score of ≤0.95. **c** Density plot showing the protein and orthogroup F-scores for all recovered genes using OrthoFiller. **d** Density plot showing the protein and orthogroup F-scores for all recovered genes in the absence of OrthoFiller filtration

Figure 3 and Table 3 show the performance statistics for OrthoFiller using a version of *S. cerevisiae* genome where 90% of gene annotations were removed (4615 annotations). This represents an extreme case where a genome has minimal annotation. The full details of detection of the deleted genes at different stages in the OrthoFiller algorithm are shown in Additional file 2: Figure S2. Again, the experiment was run 10 times, this time with disjoint subsets of 10% of genes left remaining. Here, application of OrthoFiller resulted in the identification of on average 1473 genes, of which 99.9% overlapped with the removed genes. Of the found genes, 95.2% were recovered with a protein F-score of 0.95 or greater. Of the genes with lower protein F-scores (Fig. 3b), only 4.7% had an orthogroup F-score < 0.95. As before, although these gene models differed from the original reference gene model, this difference was not sufficient to disrupt downstream orthogroup inference.

In the absence of OrthoFiller filtration, an average of 4270 genes were found, of which 97.5% overlapped the removed genes. An average of 89 removed genes were split into multiple individual genes upon recovery. On average, 90.4% of the found genes had a protein F-score ≥ 0.95. Of the genes with lower protein F-scores, 39.4% had an orthogroup F-score lower than 0.95, and 32.3% were sufficiently mis-predicted that they failed to be placed in any orthogroup at all, or in an orthogroup completely different to the one that was used to find them.

Figures  3c-d show the distribution of orthogroup F-scores versus protein F-scores for recovery in the 90% removal case. Figure 3c shows that most genes were recovered well, with 89.8% genes predicted correctly and placed in the correct orthogroup when subject to orthogroup inference (protein F-score ≥ 0.95, orthogroup F-score ≥ 0.95). Interestingly, there are many genes that are predicted correctly but are placed into a slightly different orthogroup to what was expected. This is due to changes in orthogroup membership caused by the many still-missing genes.

Thus, although the input datasets are dramatically different the performance characteristics of OrthoFiller on the 10 and 90% datasets are broadly consistent (e.g. 30.8 and 31.9% recovery of removed genes respectively, of which 94.1 and 89.8% were high-accuracy predictions).

### Evaluation of OrthoFiller on *A. thaliana* after removal of 10% of gene annotations

**Fig. 4:**
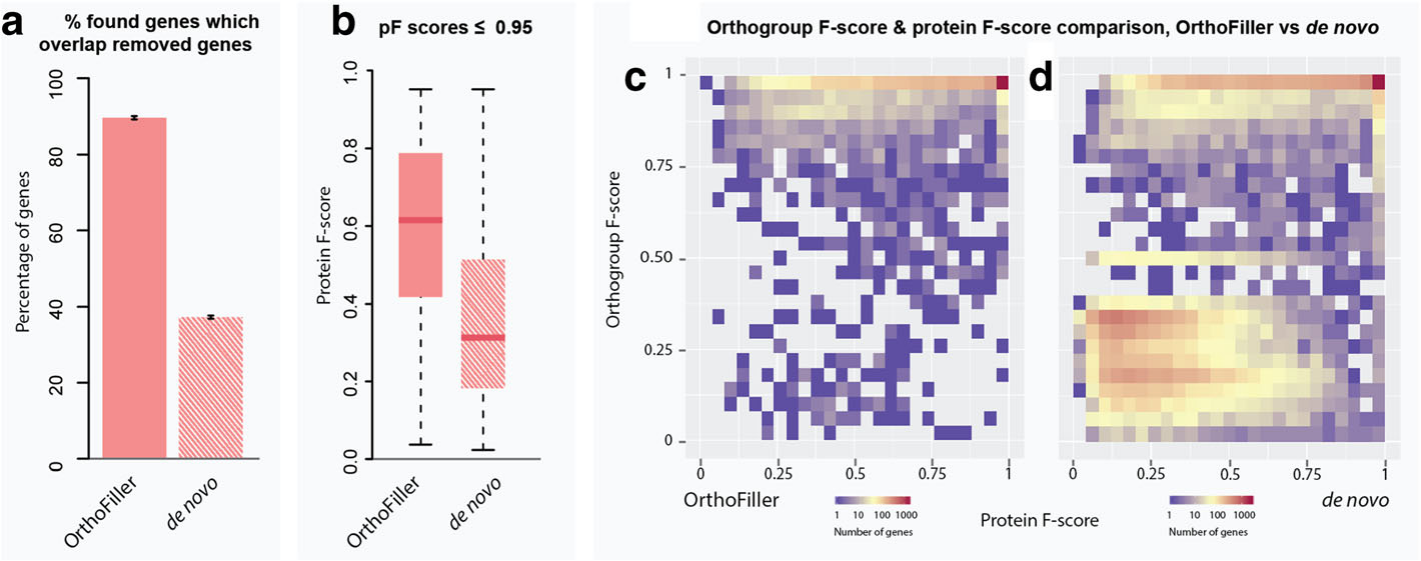
Performance of OrthoFiller on *A. thaliana* genome with 10% of annotated genes removed. **a** Using OrthoFiller 89% of found genes overlapped removed gene annotations, compared with 37% without filtration; **b** Boxplot of protein F-scores for genes predicted using OrthoFiller, versus predictions made in the absence of OrthoFiller filtration, that had a protein F-score of ≤0.95. Scores were typically lower for the *de novo* runs. **c** Density plot showing the protein and orthogroup F-scores for all recovered genes using OrthoFiller. **d** Density plot showing the protein and orthogroup F-scores for all recovered genes in the absence of OrthoFiller filtration

As it could be argued that fungal genomes present an easier challenge, an additional demonstration of the utility of OrthoFiller on an alternative group of organisms was also conducted. Here the analogous test of the method was applied to a set of five land plant genomes (Table 2). OrthoFiller was run five times with 10% of discoverable *A.* thaliana genes removed, with a different disjoint random subset removed each time. Table 4 and Fig. 4 show average performance statistics from these runs.

**Table 4:** Recovery of removed genes in A*. thaliana*

	10% annotations removed				90% annotations removed			
	OrthoFiller		*de novo*		OrthoFiller		*de novo*	
No. genes removed	2410		2410		21683		21683	
Total genes found^a^	1233	37.5	13184	426.7	11480	96.2	42504	223.5
Found genes which overlap removed genes^a^	1107	37.5	4918	130.4	11344	89.0	35609	149.5
Total recovered genes^a^	1036	31.72	2269	16.4	10380	59.7	20431	33.0
Number of split genes^a^	67	5.4	1214	23.6	945	34.4	7451	37.7
Mean pF-score of found genes^a^	0.75	0.01	0.45	(<0.01)	0.70	(<0.01)	0.55	(<0.01)
Mean oF-score of found genes^a^	0.94	(<0.01)	0.51	(<0.01)	0.89	(<0.01)	0.64	(<0.01)
High-quality found genes (pF-score ≥0.95)^a^	432.0	29.3	640.8	27.4	3419.8	21.3	9079.6	99.5
Lower-quality found genes (pF-score <0.95)^a^	674.6	32.1	4277.2	147.8	7923.8	99.5	26529.8	201.3
Mean pF-score of lower-quality genes^a^	0.61	0.02	0.31	(<0.01)	0.57	(<0.01)	0.33	(<0.01)
% of lower-quality genes with oF-score < 0.95^a^	34.6	1.9	81.1	0.9	43.4	0.6	73.9	0.1

^a^Numbers shown are rounded mean values from ten disjoint removed subsets of genes, with standard deviations bracketed

An average of 1233 genes were discovered by OrthoFiller, 89.7% of which overlapped removed genes. Of these, 39.0% were recovered to very high accuracy (protein F-score ≥ 0.95), and the mean protein score of the lower-quality genes was 0.61. 95% of these lower-quality genes were placed in the correct orthogroup after orthogroup inference, indicating that the lower protein scores were typically a product of inaccurate gene model rather than the gene itself being incorrect, and orthogroup inference remained reliable.

In the absence of OrthoFiller filtration, an average of 13184 genes were discovered, considerably more than the number of genes removed, indicating that a large number of these may be erroneous: only 37.3% of these genes overlapped removed genes. Of those that overlapped removed genes, 24.7% represented removed genes that were split into multiple parts, and only 13.0% were high quality. The average protein F-score of the lower-quality genes was 0.31, and 81.1% of these lower-quality genes also had a low (<0.95) orthogroup F-score. Thus in the absence of OrthoFiller filtration, large numbers of unreliable genes were found.

Figure 4c-d show the distribution of orthogroup F-scores versus protein F-scores for recovery in the 10% removal case for *A. thaliana*. Using OrthoFiller, 37.1% of genes had both a high (≥0.95) protein and orthogroup F-score. Furthermore, in the *de novo* case, 52.4% of recovered genes scored poorly on both metrics (<0.5), compared with 1.4% of genes found using OrthoFiller. Thus using OrthoFiller reduces the proportion of found genes which are erroneous.

### Evaluation of OrthoFiller on *A. thaliana* after removal of 90% of gene annotations

**Fig. 5:**
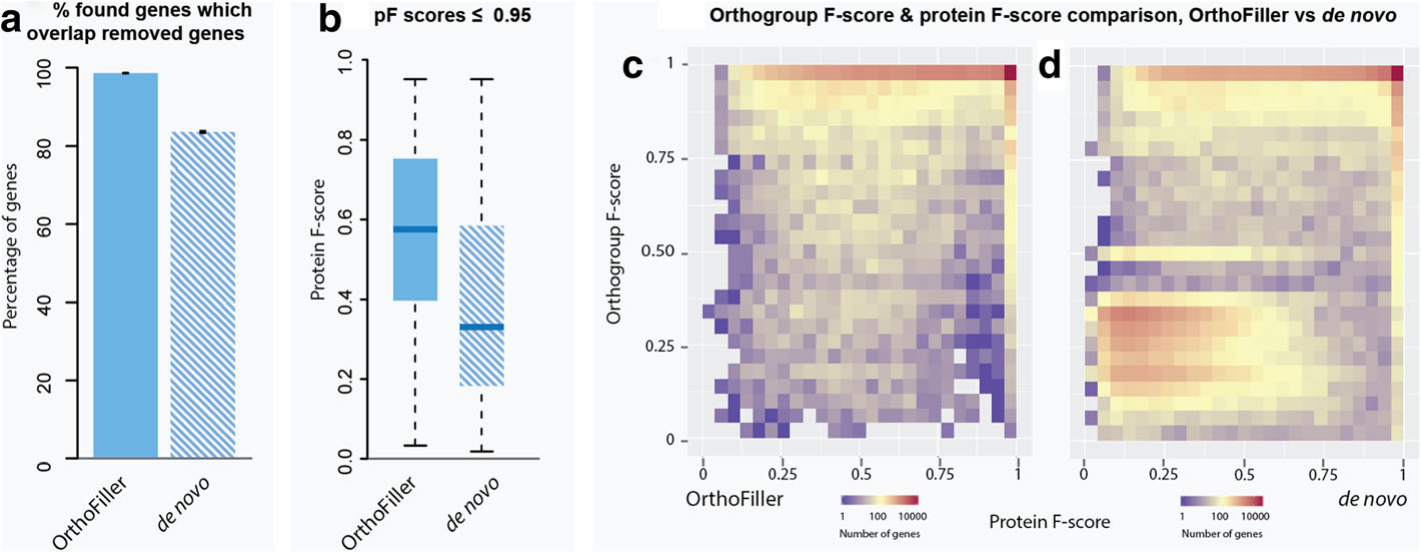
Performance of OrthoFiller *on A.* thaliana genome with 90% of annotated genes removed. **a** Using OrthoFiller 98.8% of found genes overlapped removed gene annotations, compared with 83.7% without filtration; **b** Boxplot of protein F-scores for genes predicted using OrthoFiller,  versus predictions made in the absence of OrthoFiller filtration, that had a protein F-score of ≤0.95. **c** Density plot showing the protein and orthogroup F-scores for all recovered genes using OrthoFiller. **d** Density plot showing the protein and orthogroup F-scores for all recovered genes in the absence of OrthoFiller filtration

Average performance statistics for the application of OrthoFiller to the 90% depleted *A. thaliana* genome (21683 genes removed) can be seen in Table 4 and Fig. 5. Statistics are averaged over five runs using different disjoint subsets of discoverable genes left in the annotation.

Using OrthoFiller, 11480 genes were discovered, of which 98.8% overlapped genes that had been removed. Of these, 30.1% had a high protein F-score, and the average protein F-score for those of lower quality remained relatively high at 0.57. 56.5% of genes with lower protein F-scores ended up in the right orthogroup after orthogroup inference.

In the absence of OrthoFiller filtration, many more genes were found than removed, with an average of 42504 genes found. Of these, 83.8% overlapped removed genes, however many removed genes (20%) were split into multiple parts. Only 25% of the overlapping genes were recovered to high accuracy (protein F-score ≥ 0.95), and the average protein F-score for the lower-quality genes was low at 0.33, indicating that a large number of these genes bore little resemblance to the removed gene they overlapped. In addition, 73.9% of lower-quality genes had low-quality orthogroup inference, indicating that the proteins were sufficiently mis-predicted that they disrupted orthogroup inference. This shows that, although more genes were recovered in the absence of OrthoFiller filtration, considerably more noise and erroneous predictions are produced.

Figure 5c-d show the distribution of orthogroup F-scores versus protein F-scores for recovery in the 90% removal case for *A. thaliana*. On average, 38.1% of genes recovered without OrthoFiller were of dually low quality (protein F-score, orthogroup F-score <0.5), compared with only 3.4% of genes recovered with OrthoFiller. Thus, similar to the fungal data set analysis, the performance characteristics of OrthoFiller on the 10 and 90% plant datasets are broadly consistent (e.g. 42.9 and 47.9% recovery respectively, 39.0 and 30.1% high-accuracy recoveries respectively), and both contain a considerably smaller proportion of erroneous genes than would be found without filtering.

### Evaluation of OrthoFiller on *S. cerevisiae* and *A. thaliana* with different species sets

In order to test the behaviour of OrthoFiller under different species subsets, the algorithm was run on expanded and reduced versions of species sets A and B. The species were selected to test the sensitivity of OrthoFiller to inclusion of additional close and distant relatives, and to test the behaviour of the method when different numbers of species are provided. In each case, three additional species were chosen. One species was an in-group to the original species set and the other two were progressively more distantly related out-groups to the original set. The additional species are listed in Tables 5 and 6 as species sets A+ and B+ respectively.

**Table 5:** Species Set A+, additional fungal species used for algorithm validation

Species name	Source	Strain	Taxonomy ID	References
*Aspergillus nidulans*	*JGI*	FGSC A4	227321	[27]
*Schizosaccharomyces pombe*	JGI	972 h-	4896	[28, 29]
*Zygosaccharomyces rouxii*	JGI	CBS732	559307	[30]

**Table 6:** Species Set B+, additional plant species used for algorithm validation

Species name	Source	Version	Taxonomy ID	References
*Brassica oleraceae capitata*	JGI	v1.0	3716	[31]
*Citrus clementina*	JGI	V1.0	85861	[32]
*Populus trichocarpa*	JGI	v3.1	3694	[33]

**Fig. 6:**
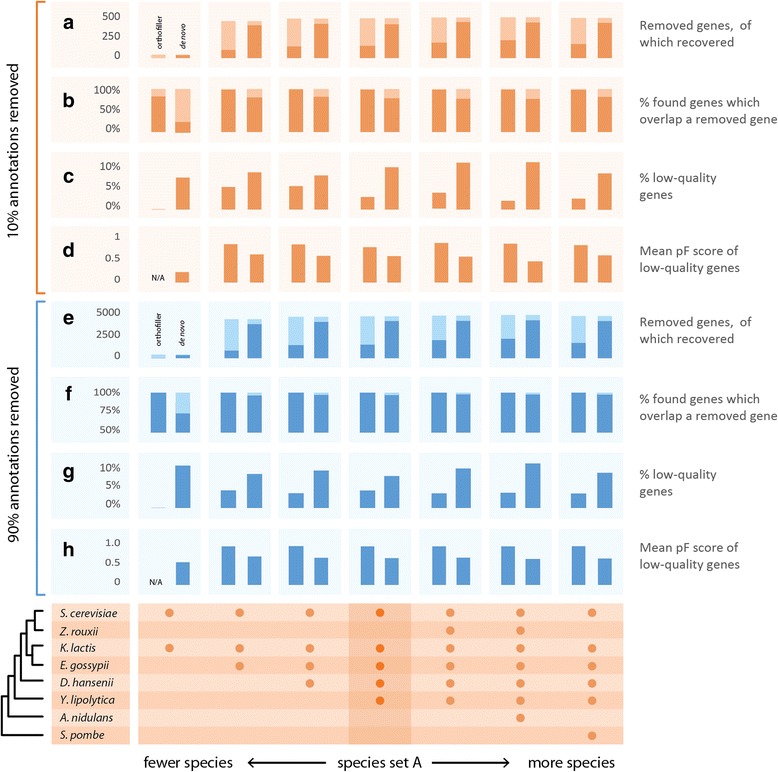
Analysis of OrthoFiller on different subsets of fungal species. Species subsets are indicated by *bullets* on the bottom panel. Behaviour of OrthoFiller is consistent across different numbers and relatedness of species, with the exception of when only two species are used. **a**, **e** Number of genes removed from *S. cerevisiae* (*light* + *darker colour*), and the proportion recovered (*darker colour*). **b**, **f** Proportion of genes found which overlap removed genes (*darker colour*) is consistently higher for OrthoFiller than for its *de novo* counterpart. **c**, **g** Percentage of recovered genes of low quality, i.e. with protein F-score <0.95, is consistently lower in OrthoFiller than the *de novo* version. **d**, **h** Mean protein F-score of lower-quality genes is consistently higher for OrthoFiller than for the *de novo* version

**Fig. 7:**
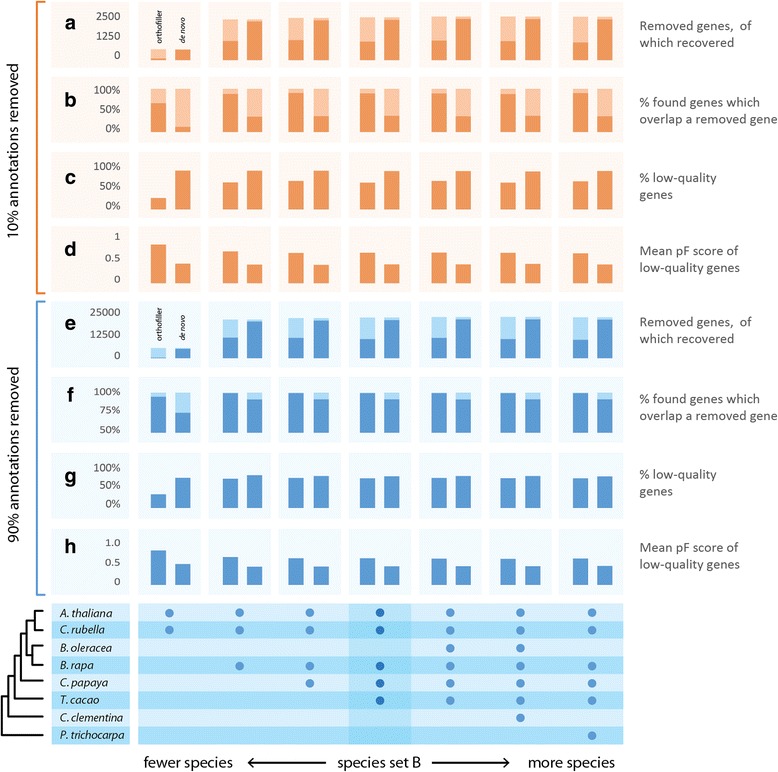
Analysis of OrthoFiller on different subsets of plant species. Species subsets are indicated by *bullets* on the bottom panel. Behaviour of OrthoFiller is consistent across different numbers and relatedness of species, with the exception of when only two species are used. **a**, **e** Number of genes removed from *A. thaliana* (*light* + *darker colour*), and the proportion recovered (*darker colour*). **b**, **f** Proportion of genes found which overlap removed genes (*darker colour*) is consistently higher for OrthoFiller than for its *de novo* counterpart. **c**, **g** Percentage of recovered genes of low quality, i.e. with protein F-score <0.95, is consistently lower in OrthoFiller than the *de novo* version. **d**, **h** Mean protein F-score of lower-quality genes is consistently higher for OrthoFiller than for the *de novo* version

For both the plant and fungal datasets, the behaviour of OrthoFiller was robust to the inclusion of additional species (Figs. 6 and 7). However, in both cases the method performed poorly when only two species were included. It is therefore recommended that users include at least three species in their analysis.

### Evaluation of the SGD reliability classification of recovered genes

The genes recovered in *S. cerevisiae* when applying OrthoFiller after 10 and 90% removal were compared with reliability classifications provided by the SGD. In this genome version, the SGD classified 5103 genes as verified, 784 as dubious, and 806 as uncharacterised. Of the 4766 verified genes that were clustered into orthogroups and hence discoverable by OrthoFiller, 1857 genes were recovered. Of the dubious genes, only three were clustered into orthogroups, and none of these were recovered by OrthoFiller. Of the 806 uncharacterised genes, 357 were clustered into orthogroups, and 53 were recovered by OrthoFiller. Thus dubious and uncharacterised genes are recovered at lower rates than verified genes.

### OrthoFiller detects hundreds of conserved genes not present in the reference genome annotations

In addition to testing the ability of OrthoFiller to recover already predicted genes, the algorithm was applied to both of the sets of complete genomes listed in Tables 1 and 2, to assess the potential for novel genes to be discovered. In total, 34 novel genes were found across the fungal species evaluated, and 830 novel genes were found across the plant species. The number of genes found for each species in each set is listed in Tables 7 and 8. All newly predicted genes described in this study are publicly available for download from the Zenodo Research Data Repository (see Implementation).

**Table 7:** Novel genes in fungal species

Species name	Genome size (Mbp)	No. pre-existing genes	No. new genes.
*E. gossypii*	9.10	4768	2
*D. hansenii*	12.15	6272	13
*K. lactis*	10.69	5076	8
*S. cerevisiae*	12.16	6572	2
*Y. lipolytica*	20.50	6447	9

**Table 8:** Novel genes in plant species

Species name	Genome size (Mbp)	No. pre-existing genes	No. new genes.
*A. thaliana*	119.67	27416	116
*B. rapa*	315.05	40492	10
*C. papaya*	342.68	27751	382
*C. rubella*	134.83	26521	228
*T. cacao*	346.16	29452	94

**Fig. 8:**
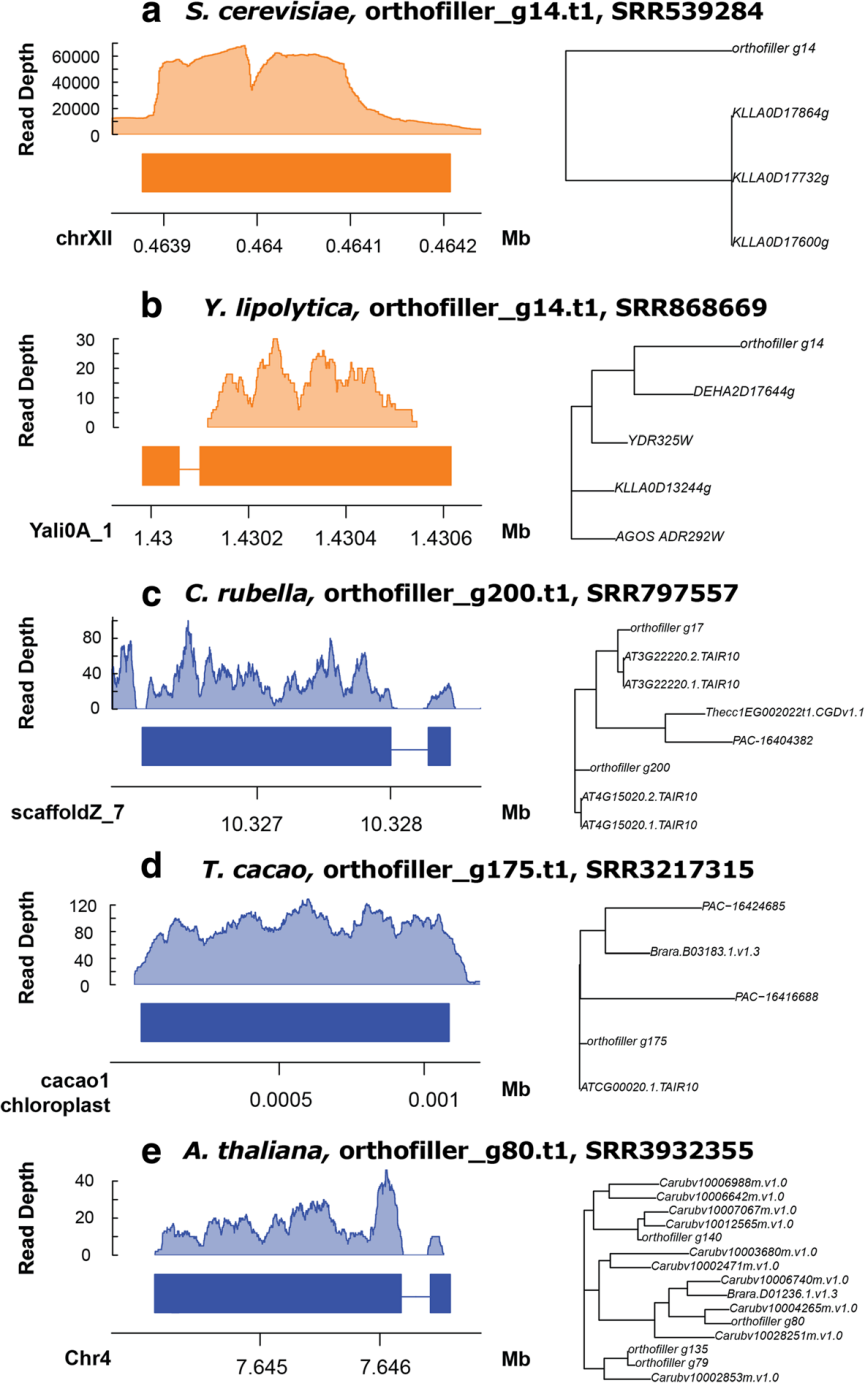
Five representative examples of RNAseq coverage on genes predicted using OrthoFiller. Coverage plots demonstrate RNAseq reads mapping to locations of new genes, and phylogenetic trees demonstrate the relationship of the newly predicted gene to other genes in the orthogroup. **a **Species *S. cerevisiae*, gene orthofiller_g14.t1, RNAseq data from SRR539284. **b **Species *Y. lipolytica*, gene orthofiller_g14.t1, RNAseq data SRR868669. **c **Species* C. rubella*, gene orthofiller_g200.t1, RNAseq data SRR797557. **d **Species *T. cacao*, gene orthofiller_g175.t1, RNAseq data SRR321315. **e **Species *A. thaliana*, gene orthofiller_g80.t1, RNAseq data SRR3932355

To be detected as a novel gene OrthoFiller requires genes to pass rigorous sequence similarity tests to genes in other species (including empirical evaluation of sequence similarity scores to distinguish real from spurious hits), which in itself provides evidence for the existence of predicted genes through homology. To provide additional evidence for the existence of the novel predicted genes they were subjected to analysis using publicly available RNAseq data from the Sequence Read Archive (SRA) [16]. The datasets used for this analysis are listed in Tables 9 and 10, and examples of RNAseq coverage on a selection of novel genes can be found in Fig. 8. The tables also show the percentage of the novel genes found that had evidence for their existence in the RNAseq data. For most genomes, most genes predicted by OrthoFiller are highly supported by RNAseq evidence, with the all the novel fungal genes exhibiting RNAseq evidence, and on average 67.2% of novel plant genes being supported. Given that the plant RNAseq datasets come from single tissue samples under a single condition it is not expected that all genes will be detected in these samples. For example, similar detection statistics were obtained for the original predicted genes from the source datasets, shown in Tables 9 and 10. It should also be noted that genes that are present in RNAseq data are more likely to have been annotated already, given that many genome annotation pipelines rely on such data to perform their analyses [17].

**Table 9:** SRA RNA-seq data coverage for novel genes in fungal genomes

Species	SRA ID	Instrument/details	Genes in original annotation			Novel genes		
			Total	W/ reads	%	Total	W/ reads	%
*E. gossypii*	*N/A* ^a^	*N/A*	4768	*N/A*	*N/A*	*2*	*N/A*	*N/A*
*D. hansenii*	SRR1296968	Illumina HiSeq 2000, paired end	5781	6272	92.2%	13	13	100%
*K. lactis*	SRR1200528	Illumina Genome Analyzer II, single	5075	5076	100%	8	8	100%
*S. cerevisiae*	SRR539284	Illumina HiSeq 2000, paired end	6560	6572	99.8%	2	2	100%
*Y. lipolytica*	SRR868669	Illumina HiSeq 2000, single	6432	6447	99.8%	9	9	100%

^a^No publically available data found for this species/strain

**Table 10:** SRA RNA-seq data coverage for novel genes in plant genomes

Species	SRA ID	Instrument/details	Genes in original annotation			Novel genes		
			Total	W/ reads	%	Total	W/ reads	%
*A. thaliana*	SRR3932355	Illumina HiSeq 2500, paired end. Wild type Columbia rep1	27416	26110	95.2	116	89	76.7
*B. rapa*	SRR2984945	Illumina HiSeq 2000, paired end. ga-deficient dwarf (gad1-2) + GA rep2	40492	35793	88.4	10	4	40.0
*C. papaya*	SRR3509576	Illumina HiSeq 2500, paired end. SunUp/Sunset cultivar, young hermaphrodite leaf	27751	24589	88.6	382	327	85.6
*C. rubella*	SRR797557	Illumina Genome Analyzer IIx, paired end	26521	21239	80.1	228	137	60.1
*T. cacao*	SRR3217315	Illumina HiSeq 2000, paired end. Flower/leaf sample	29452	25758	87.5	94	69	73.4

## Discussion

Here we present OrthoFiller, an automated method for improving the completeness of genome annotations. It leverages information from multiple taxa, clustering genes into orthogroups and finding genes that are conserved between species but that have escaped detection. OrthoFiller is designed to be stringent, conservatively identifying genes that can be confidently identified as missing members of existing orthogroups. Specifically, to pass the filtration criteria for detection by OrthoFiller, genes must be members of orthogroups conserved in multiple species. Thus OrthoFiller will not find genes that lack homologues in other species. These stringent criteria mean that not all genes that could be detected will be detected by the algorithm, but rather that the user should have confidence in the validity of genes identified by the method.

OrthoFiller is intended to be run after a genome annotation is considered by the user to be complete or near-complete. OrthoFiller is designed with small scale genome sequencing projects in mind and is provided to enable users without significant resources for comprehensive RNAseq-based genome annotation to leverage information from related species to improve their genome annotations. However, OrthoFiller is equally suited for use in large-scale genome comparisons, reliably filling gaps in gene sets prior to large scale comparative genomics investigations. Application of OrthoFiller in these cases will enable the downstream analysis of genes that would otherwise have been classified as absent.

The utility of OrthoFiller is demonstrated on both fungal and plant genome datasets, both in its ability to successfully find missing genes, and in the effectiveness of its filters in eliminating low-quality gene predictions. Application of this method to small groups of plant and fungal genomes resulted in the identification of 34 and 830 genes respectively. These genes are conserved in one or more species but were absent from the genome annotation in which they were predicted. We anticipate that application of OrthoFiller to larger datasets will likely result in further genome annotation improvement. Alternative publicly available genome versions for the fungal species exist such as those housed at gryc.inra.fr [18]. Although the genome annotations differ to the ones used in this study, 24 of the genes predicted by OrthoFiller were also not present in these alternative genome annotations (details available in Zenodo dataset that accompanies this manuscript, see Implementation). The quality of genes found by OrthoFiller was assessed by artificial removal and recovery of subsets of genes from a single genome, treating those original gene models as true, and evaluating the quality of those genes that were recovered by comparison to the removed genes. In the absence of the OrthoFiller filtration steps, the proportion of poor-quality genes that are recovered is considerably higher, furthermore OrthoFiller avoids the over-prediction of genes that can occur in many of its *de novo* counterparts.

OrthoFiller is mainly designed for use on genomes that have already undergone some basic level of annotation. As can be seen by comparing the 10% and 90% removal cases in the two data sets, application to very poorly annotated genomes can result in more genes of dubious quality, both from a sequence and an orthogroup perspective. It is worth noting that many of the genes with lower-quality scores, particularly those with only one of the scores being low, can be explained by alternate gene models (in the protein F-score) and shifting of orthogroups due to altered proteome sets (in the orthogroup F-score case). In all cases, in the absence of OrthoFiller filtration considerably higher numbers of genes were predicted that didn’t resemble the genes that they were supposed to, indicating that they are erroneous. The results of OrthoFiller were robust to addition or removal of species. However, OrthoFiller performed poorly when only two species were used as input. It is therefore recommended that at least three species are used as input for OrthoFiller.

The OrthoFiller algorithm is designed to run on a Unix system with python and a minimal number of standard additional tools (HMMer, BedTools, Augustus, R, OrthoFinder). The software can be downloaded from https://github.com/mpdunne/orthofiller, where installation and implementation instructions can also be found. The speed of the algorithm is principally dependent on the speed of Augustus and HMMer, however processing time can be decreased by running OrthoFiller on multiple CPUs and thus parallelising these steps of the method.

Accurate and complete genome annotation is of paramount importance to the effective analysis of genomic and transcriptomic data, as well as for phylogenetic inference from genomic data. As the quantity of published genomes increases, care must be taken to ensure accuracy and quality of genome annotations are maintained. Automated methods that leverage publicly available information from multiple species to improve the annotation of newly sequenced genomes will help improve the accuracy and completeness of these resources and thus the quality of all analyses that utilise them.

## Conclusions

OrthoFiller is an algorithm for improving the completeness of genome annotations. It leverages data from multiple species to identify conserved genes that have escaped detection, correct these detection errors and thus improve the genome completeness of all species under consideration.

## Implementation

### Data sources

For algorithm development and evaluation, a set of five well-annotated fungal genomes (Table 1) and a set of five well-annotated plant genomes (Table 2) were selected. Evaluation of the algorithm focussed on *S. cerevisiae* and *A. thaliana*, as the gene models in these genomes have historically been subject to extensive improvement and revision and are the most likely to be correct.

### Algorithm overview

OrthoFiller proceeds in five stages summarised in Fig. 1 and described in detail in the following sections. In brief, the algorithm begins by inferring a set of orthogroups from the protein coding genes of the set of species submitted to OrthoFiller (Fig. 1a). The protein sequences in these orthogroups are subject to multiple sequence alignment, converted to nucleotide sequences and used to build HMMs. These HMMs are used to search the genomes of each species under consideration (Fig. 1b) and the resultant HMM hits are subject to stringent filtering (Fig. 1c) before being used as hints for gene model construction (Fig. 1d). The gene models are subject to additional filtering (Fig. 1e) and only those gene models that pass all filters are added to the revised genome annotation. The revised genome annotations are then subject to orthogroup inference (Fig. 1f) and resultant orthogroups are analysed to confirm the identity of the newly predicted genes. The complete details for each step of this algorithm are described in the sections below.

### Inference of Orthogroups and construction of HMMs

Orthogroups are inferred using OrthoFinder [10]. If a gene from the source annotation is not included in an orthogroup with at least one other sequence, it is classed as a *singleton*, and is not considered in downstream analyses. This is consistent with the problem definition of OrthoFiller, that is to identify unannotated genes that are conserved between species. Amino acid sequences from the orthogroups are aligned with MAFFT [19], using the L-INSI algorithm, and the resultant multiple sequence alignments are back-translated using the source nucleotide sequences. Aligning in this way, i.e. by amino acid sequence first, increases the robustness of the alignment under synonymous mutations and thus provides higher alignment quality [20]. The resulting nucleotide alignments are converted to Hidden Markov models (HMMs) using HMMer [21], each of which is then searched against each input genome in turn to generate a set of hits per HMM per species.

### Evaluation of HMM search results

Due to the probabilistic nature of HMM searches, there is considerable variation in the quality of the relationship between a hit region and the set of sequences used to generate the source HMM. One expects a large amount of “background noise”, that is sequence regions which pass the thresholds of the HMM but whose relevance is dubious. Each HMM hit has an associated bit score, an aggregated base-by-base similarity score between the hit and the aligned sequences used to generate it: we use this score to assess the quality of the hit. The bit score is strongly dependent on the hit length, thus to prevent gene length from biasing downstream analyses the bit score of a hit is divided by the hit length, to generate the *adjusted score* for a hit *h:*

$$ scor{e}_{adj}(h)=\frac{score(h)}{length(h)} $$

The adjusted score is related to the e-value. However, the e-value calculation enforces a strict lower limit of $\sim 1\times {10}^{-300}$, all lower scores being rounded down to zero. Thus use of e-values would introduce irreversible length bias and would lead to downstream errors, as has been shown previously [10]. As bit scores do not have a threshold value, and they have been previously shown to be capable of facilitating accurate inference of phylogenetic trees [22], and length-corrected bit scores are used as the basis of the scoring scheme in OrthoFinder [10], they were used here.

For each species, a threshold value for hit acceptance or rejection based on a hit’s adjusted score is created, by considering the distribution of hits which overlapped known genes. Anything above this threshold is considered to be genuine, and anything below this threshold is considered to be noise. An HMM hit is classed as *good* if it overlaps any gene from the orthogroup used to create the HMM, *bad* if it only overlaps genes from orthogroups other than the one used to create the HMM, and *candidate* if it overlaps no known gene at all. Here candidate hits are the potential new genes of interest, and the *good* and *bad* genes are used to inform our judgement about the reliability of the candidate hits.

Distributions of adjusted scores for good and bad hits to the *S. cerevisiae* genome from all HMMs generated by the species in Table 1 are provided as an example in Additional file 3: Figure S3. Distributions for good and bad hits are clearly delineated into two distinct distributions. Note that in this case there are relatively few candidate hits, since the genome under inspection is already well annotated and is expected to have few missing gene predictions. Skew-t distributions are fit separately to the good and bad score distributions using *gamlss* [23]. Skew distributions were chosen because they allow flexibility in location, shape and scale of the underlying data and are commonly used for estimating parameters such as location and scale, while allowing the same distribution type to be used to fit both the good and bad hits. A separate skew-t distribution for the good and bad hits is fit for each species. In the event that there are insufficient good and bad hits to fit distributions, good and bad hits from the other species are aggregated and a threshold value is calculated from this.

For a given adjusted score $x$, the distributions of the *good* and *bad* hits are used to estimate both the absolute probabilities of a hit being genuine or being a mistake. We can estimate

$$ P\left( genuine\Big| x\right) = \frac{P\left( x\Big| genuine\right) P(genuine)}{P\left( x\Big| genuine\right) P(genuine)+ P\left( x\ \Big| mistake\right) P(mistake)} $$

$$ P\left( mistake\Big| x\right) = \frac{P\left( x\Big| mistake\right) P(mistake)}{P\left( x\Big| genuine\right) P(genuine)+ P\left( x\ \Big| mistake\right) P(mistake)} $$

and then retain the hit depending on whether it has a higher probability of being genuine than being a mistake, based on its adjusted score. The probabilities $P(genuine)$ and $P( m i s t a k e)$ are estimated by considering the proportion of good/bad hits which are good and bad respectively. The probability density functions $P\left( x\right| genuine\Big)$ and $P\left( x\Big| mistake\right)$ are determined using the fitted distributions as described above.

### Acquisition and evaluation of putative predicted genes

Hits which survive the hit filtration step are passed to the gene-finding program Augustus [11] as *hints* specified as exon parts. Only predicted genes that have a nonzero overlap with these hints are retained. These predicted genes are then subjected to a *hint filter*, which aims to separate those genes which have genuinely arisen from the hint from those that overlap the hint by chance. The hint filter evaluates a *hint F-score* for each predicted gene, by comparing against the hints from a particular orthogroup which overlap it. The hint F-score is a measure of how well the found gene corresponds to the hints used to inform its discovery. Each predicted gene *G* will have at least one *hint region* corresponding to it, which is a set of non-overlapping coordinates obtained from merging all hints that overlap *G*, and which are all derived from the same orthogroup. For a hint region *H* and a predicted gene *G*, the hint F-score is defined as:

$$ h f\left( H,\  G\right)=\frac{2\cdot h P\left( H, G\right)\cdot h R\left( H, G\right)}{hR\left( H, G\right)+ hP\left( H, G\right)} $$

where

$$ h P\left( H, G\right)=\frac{\left| H\cap G\right|}{\left| H\right|};\  h R\left( H, G\right)=\frac{\left| H\cap G\right|}{\left| G\right|} $$

The filter uses a threshold hint F-score value of 0.8 (i.e. on average 80% of the length of the predicted gene is covered by the hit and vice versa), below which potential gene models are discarded. This value was chosen based on an analysis of hint F-scores of good and bad hits (as defined above) versus the Augustus output corresponding to them. Example distributions for hint F-scores for the good and bad hits can be seen in Additional file 4: Figure S4, in which it can be clearly seen that practically all genuine hints pass the threshold value of 0.8.

Once gene models have been filtered, they are fed once again into OrthoFinder, to cluster them into orthogroups. The orthogroup of each newly predicted gene is compared with the orthogroup(s) which were used to predict that gene. It is possible that multiple orthogroups informed the prediction of the same gene; similarly, there may be fluctuations in orthogroup membership between the original and new genomes. It is therefore only required that the new orthogroup into which the gene is clustered has non-zero overlap with at least one of the orthogroups used to predict it, and genes which do not fulfil this criterion are discarded.

### Algorithm evaluation

#### Recovery of removed genes

The test set of species from Table 1 was used to analyse the effectiveness of OrthoFiller for genomes of various levels of completion. Altered versions of the *S. cerevisiae* genome annotation were constructed with 10 and 90% of genes randomly removed, and the level of recovery of the removed genes upon implementation of OrthoFiller was assessed, where a gene G was considered to be *recovered* if OrthoFiller predicted a gene such that G and G’ have non-zero overlap.

The quality of the predicted genes was assessed by considering two scores: the orthogroup F-score and the protein F-score. The protein F-score is defined as

$$ p F\left( S, S{}^{\prime}\right)=\frac{2\cdot p P\left( S,{S}^{\prime}\right)\cdot p R\left( S,{S}^{\prime}\right)}{pR\left( S,{S}^{\prime}\right)+ pP\left( S,{S}^{\prime}\right)} $$

where $S$ is the original amino acid sequence and $S^{\prime }$ is the amino acid sequence of the recovered gene, and

$$ p P\left( S,{S}^{\prime}\right)=\frac{\left| S\cap {S}^{\prime}\right|}{\left| S\right|}; p R\left( S,{S}^{\prime}\right)=\frac{\left| S\cap {S}^{\prime}\right|}{\left|{S}^{\prime}\right|} $$

where the sequence length is the number of amino acids in the sequence, and the intersection length is defined to be the sum of identical amino acids in an alignment (MAFFT L-INSI) of the two sequences. The orthogroup F-score is defined as

$$ o F\left( S,{S}^{\prime}\right)=\frac{2\cdot o P\left( O,{O}^{\prime}\right)\cdot o R\left( O,{O}^{\prime}\right)}{oR\left( O,{O}^{\prime}\right)+ oP\left( O,{O}^{\prime}\right)} $$

where $O$ is the orthogroup that the gene is placed when no deductions have been made, $O^{\prime }$ is the orthogroup into which the gene is placed when OrthoFinder is run on the OrthoFiller results, and

$$ o P\left( O,{O}^{\prime}\right)=\frac{\left| O\cap {O}^{\prime}\right|}{\left| O\right|}; o R\left( O,{O}^{\prime}\right)=\frac{\left| O\cap {O}^{\prime}\right|}{\left|{O}^{\prime}\right|} $$

where cardinality of the orthogroups takes into account only genes which were present in the input set of genome annotations, i.e. not counting the newly discovered genes.

### Evaluation of novel predicted genes

RNA-seq data was downloaded from the Sequence Read Archive, and aligned to the genome with HISAT2 [24] using default parameters. Coverage was calculated using BedTools coverage [25].

## Availability and requirements

The software is available under the GPLv3 licence at https://github.com/mpdunne/orthofiller. All newly predicted genes and gene models are available for download from the Zenodo Research Data Repository doi.org/10.5281/zenodo.546090.

## Additional files

Additional file 1: Figure S1. Recovery of removed genes from *S. cerevisiae* after 10% removal: Representation of removed genes at each stage, OrthoFiller vs. *de novo*. **A**) The number of deleted genes that obtained hits from one or more orthogroup HMMs. **B**) The number of deleted genes that had hits after OrthoFiller hint filtration. **C**) No hint filtration. **D**) The number of deleted genes for which a gene prediction was made using Augustus that satisfied OrthoFiller filtration tests. **E**) The number of deleted genes that for which a gene prediction was made using Augustus in the absence of OrthoFiller filtration. **F**) The number newly predicted genes that were retained or discarded based on the orthogroup assignment filter step in OrthoFiller. (TIF 562 kb)

Additional file 2: Figure S2. Recovery of removed genes from *S. cerevisiae* after 90% removal: Representation of removed genes at each stage, OrthoFiller vs. *de novo*. **A**) The number of deleted genes that obtained hits from one or more orthogroup HMMs. **B**) The number of deleted genes that had hits after OrthoFiller hint filtration. **C**) No hint filtration. **D**) The number of deleted genes for which a gene prediction was made using Augustus that satisfied OrthoFiller filtration tests. **E**) The number of deleted genes for which a gene prediction was made using Augustus in the absence of OrthoFiller filtration. **F**) The number newly predicted genes that were retained or discarded based on the orthogroup assignment filter step in OrthoFiller. (TIF 573 kb)

Additional file 3: Figure S3. hit score distributions for *good*, *bad* and *candidate* hits. Hits are to the *S. cerevisiae* genome, using HMMs from all orthogroups from the five fungal species described in Table 1. **A**) Length normalised bit scores of HMM hits to regions of the genome that contained genes that were not part of the orthogroup used to generate the HMM (bad hits). **B**) Length normalised bit scores of HMM hits to regions of the genome that do contain the gene used to generate the HMM (good hits). **C**) Length normalised bit scores of HMM hits to regions of the genome that do not contain any previously annotated genes (candidate novel gene hits). **D**) All distributions overlaid. (TIF 143 kb)

Additional file 4: Figure S4. Example distribution of hint F-scores for good vs. bad hints. Here, Augustus has been allowed to predict genes that are already present in the input genome, hence we can consider separately the good and bad hits as hints. Hits and hints are from running OrthoFiller on the five fungal species described in Table 1. Shown are the distributions of hint F-scores for good (green) and bad (red) hits respectively, demonstrating that practically all of the genuine hints have a hint F-score of 0.8 or higher. (TIF 1424 kb)

## Abbreviations

GTF: General transfer format
HMM: Hidden Markov model
oF: orthogroup F-Score
pF: protein F-score
