# Erratum to: OrthoFiller: utilising data from multiple species to improve the completeness of genome annotations

**DOI:** 10.1186/s12864-017-3849-5

**Published:** 2017-06-27

**Authors:** Michael P. Dunne, Steven Kelly

**Affiliations:** Department of Plant Sciences, University of Oxford, South Parks Road, Oxford, OX1 3RB UK

## Erratum

After publication of the original article [1] the authors found the following errors had occurred:

1. Saccaromyces should be spelt Saccharomyces in Table 1. (Table 1)
2. Kluveromyces should be spelt Kluyveromyces in Table 1. (Table 1)
3. Table 4, columns 3, 5, 7 and 9: All values within these columns should be placed in brackets to indicate the standard deviation. (Table 4)
4. The legend for Table 4 should read: ‘Numbers shown are rounded mean values from *5* disjoint removed subsets of genes, with standard deviations bracketed and not ‘*10* removed subsets’ as per the original article. (Table 4)

**Table 1:** Species Set A, fungal species used for algorithm validation

Species Name	Source	Strain	Taxonomy ID	References
*Eremothecium gossypii*	JGI^a^	*ATCC10895*	284,811	[12]
*Debaromyces hansenii*	JGI	*CBS767*	284,592	[13] [14]
*Kluyveromyces lactis*	JGI	*CLIB210*	284,590	[13]
*Saccharomyces cerevisiae*	SGD^b^	*S288C*	559,292	[24]
*Yarrowia lipolytica*	JGI	*CLIB122*	284,591	[13]

^a^Joint Genome Institute; ^b^Saccharomyces Genome Database

**Table 4:** Recovery of removed genes in A. thaliana, averaged over five runs

	10% annotations removed				90% annotations removed			
	OrthoFiller		de novo		OrthoFiller		de novo	
No. genes removed	2410		2410		21,683		21,683	
Total genes found^a^	1233	(37.5)	13,184	(426.7)	11,480	(96.2)	42,504	(223.5)
Found genes which overlap removed genes^a^	1106	(37.5)	4918	(130.4)	11,343	(89.0)	35,609	(149.5)
Total recovered genes^a^	1035	(31.72)	2268	(16.4)	10,380	(59.7)	20,430	(33.0)
Number of split genes^a^	67	(5.4)	1213	(23.6)	944	(34.4)	7451	(37.7)
Mean pF-score of found genes^a^	0.75	(0.01)	0.45	(<0.01)	0.70	(<0.01)	0.55	(<0.01)
Mean oF-score of found genes^a^	0.94	(<0.01)	0.51	(<0.01)	0.89	(<0.01)	0.64	(<0.01)
High-quality found genes (pF-score ≥ 0.95)^a^	432.0	(29.3)	640.8	(27.4)	3419.8	(21.3)	9079.6	(99.5)
Lower-quality found genes (pF-score < 0.95)^a^	674.6	(32.1)	4277.2	(147.8)	7923.8	(99.5)	26,529.8	(201.3)
Mean pF-score of lower-quality genes^a^	0.61	(0.02)	0.31	(<0.01)	0.57	(<0.01)	0.33	(<0.01)
% of lower-quality genes with oF-score < 0.95^a^	34.6	(1.9)	81.1	(0.9)	43.4	(0.6)	73.9	(0.1)

^a^Numbers shown are rounded mean values from 5 disjoint removed subsets of genes, with standard deviations bracketed

The original article has been corrected.

Corrected versions of all figures and tables are included in this Erratum:

Corrected Table 1

Corrected Table 4
