# Supplementary figures and images for: OrthoFiller: utilising data from multiple species to improve the completeness of genome annotations
Source: BMC Genomics. 2017 May 18;18:390. doi: 10.1186/s12864-017-3771-x (PMC5437544; doi:10.1186/s12864-017-3771-x)

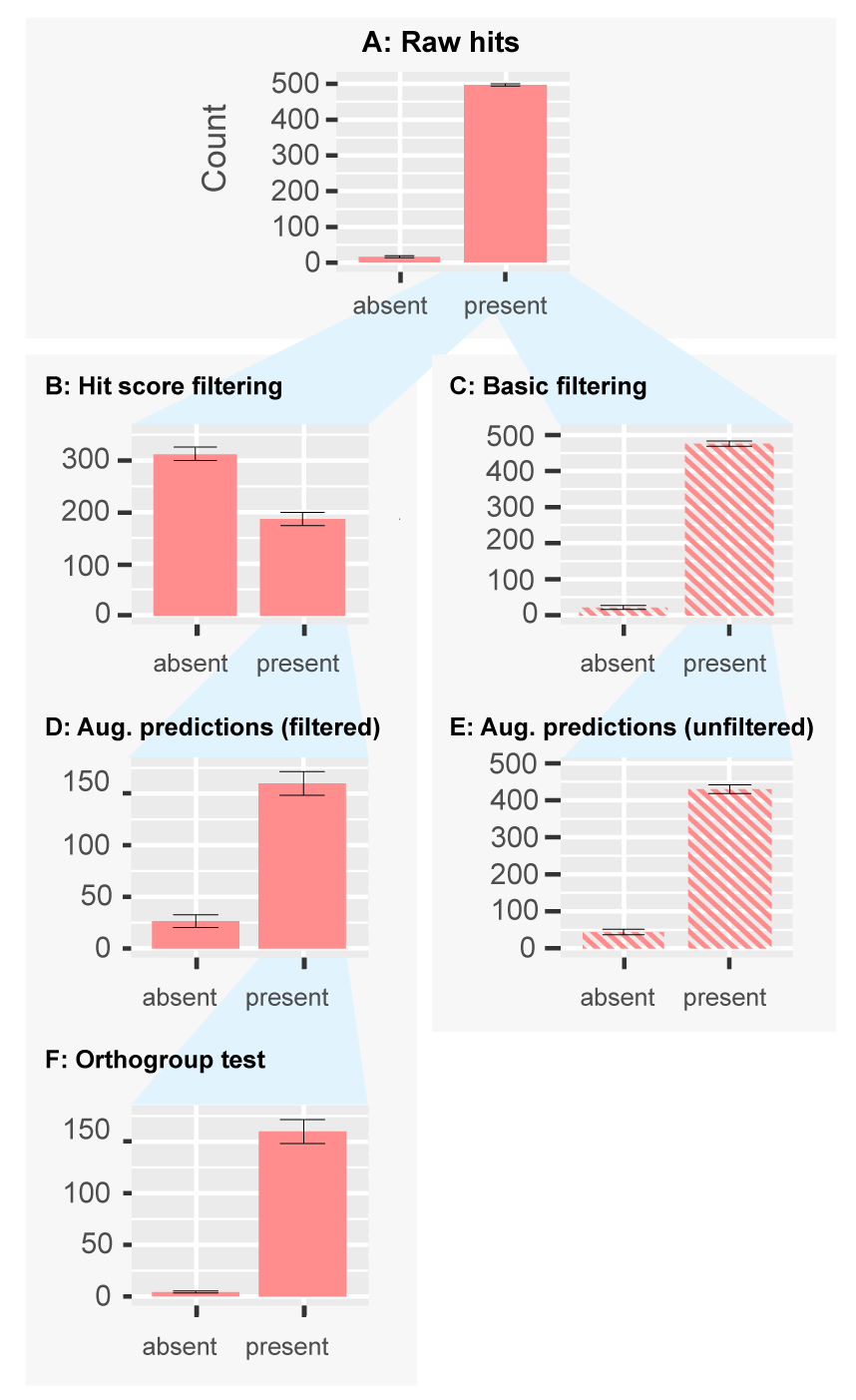

Supplement: Supplementary file 1 — Recovery of removed genes from S. cerevisiae after 10% removal: Representation of removed genes at each stage, OrthoFiller vs. de novo. A) The number of deleted genes that obtained hits from one or more orthogroup HMMs. B) The number of deleted genes that had hits after OrthoFiller hint filtration. C) No hint filtration. D) The number of deleted genes for which a gene prediction was made using Augustus that satisfied OrthoFiller filtration tests. E) The number of deleted genes that for which a gene prediction was made using Augustus in the absence of OrthoFiller filtration. F) The number newly predicted genes that were retained or discarded based on the orthogroup assignment filter step in OrthoFiller. (TIF 562 kb) [file 12864_2017_3771_MOESM1_ESM.tif]

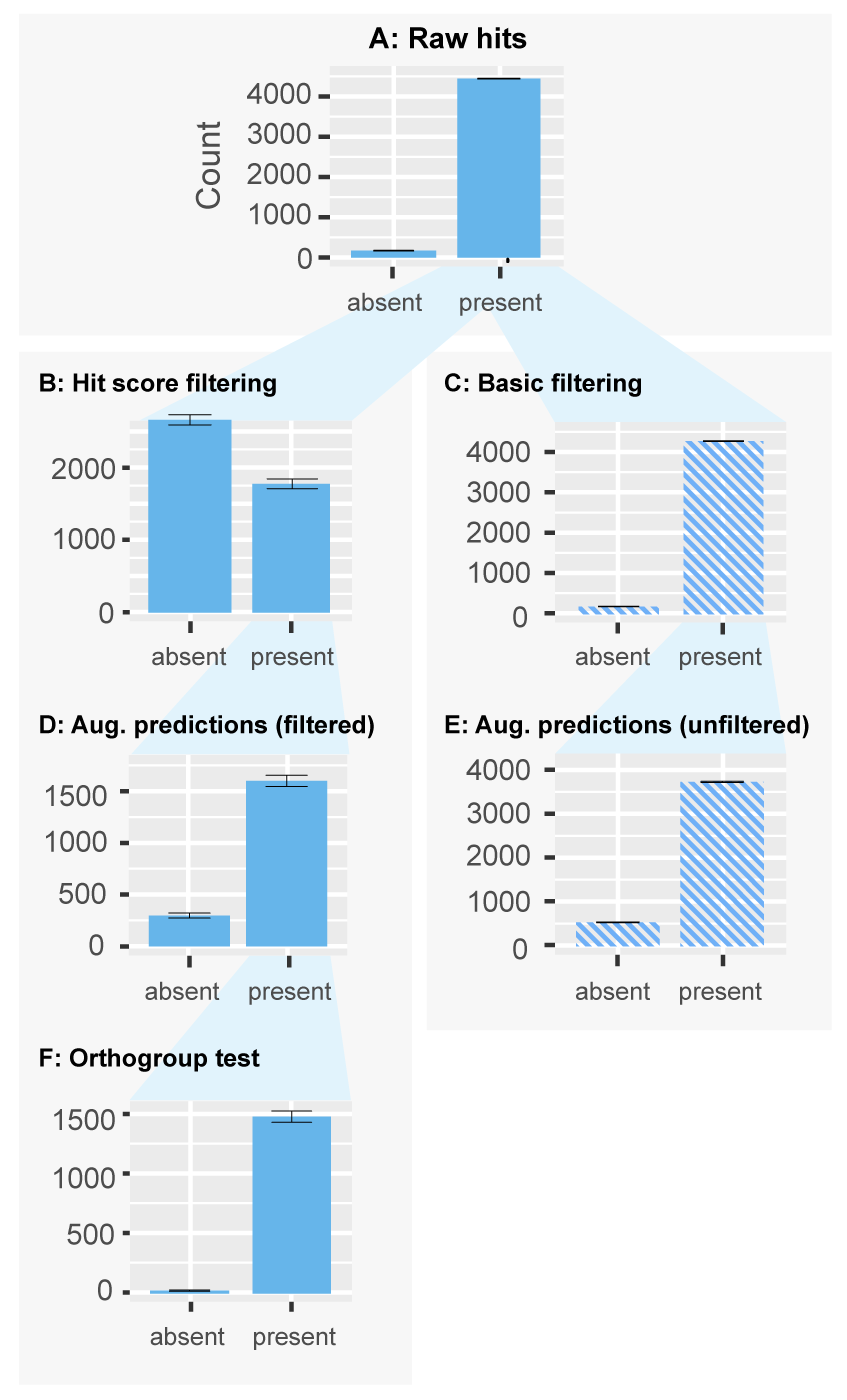

Supplement: Supplementary file 2 — Recovery of removed genes from S. cerevisiae after 90% removal: Representation of removed genes at each stage, OrthoFiller vs. de novo. A) The number of deleted genes that obtained hits from one or more orthogroup HMMs. B) The number of deleted genes that had hits after OrthoFiller hint filtration. C) No hint filtration. D) The number of deleted genes for which a gene prediction was made using Augustus that satisfied OrthoFiller filtration tests. E) The number of deleted genes for which a gene prediction was made using Augustus in the absence of OrthoFiller filtration. F) The number newly predicted genes that were retained or discarded based on the orthogroup assignment filter step in OrthoFiller. (TIF 573 kb) [file 12864_2017_3771_MOESM2_ESM.tif]

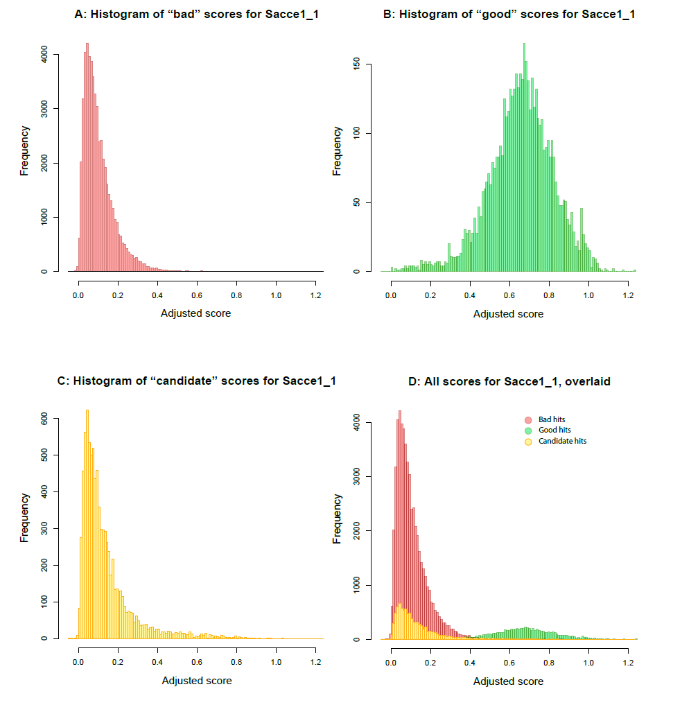

Supplement: Supplementary file 3 — hit score distributions for good, bad and candidate hits. Hits are to the S. cerevisiae genome, using HMMs from all orthogroups from the five fungal species described in Table 1. A) Length normalised bit scores of HMM hits to regions of the genome that contained genes that were not part of the orthogroup used to generate the HMM (bad hits). B) Length normalised bit scores of HMM hits to regions of the genome that do contain the gene used to generate the HMM (good hits). C) Length normalised bit scores of HMM hits to regions of the genome that do not contain any previously annotated genes (candidate novel gene hits). D) All distributions overlaid. (TIF 143 kb) [file 12864_2017_3771_MOESM3_ESM.tif]

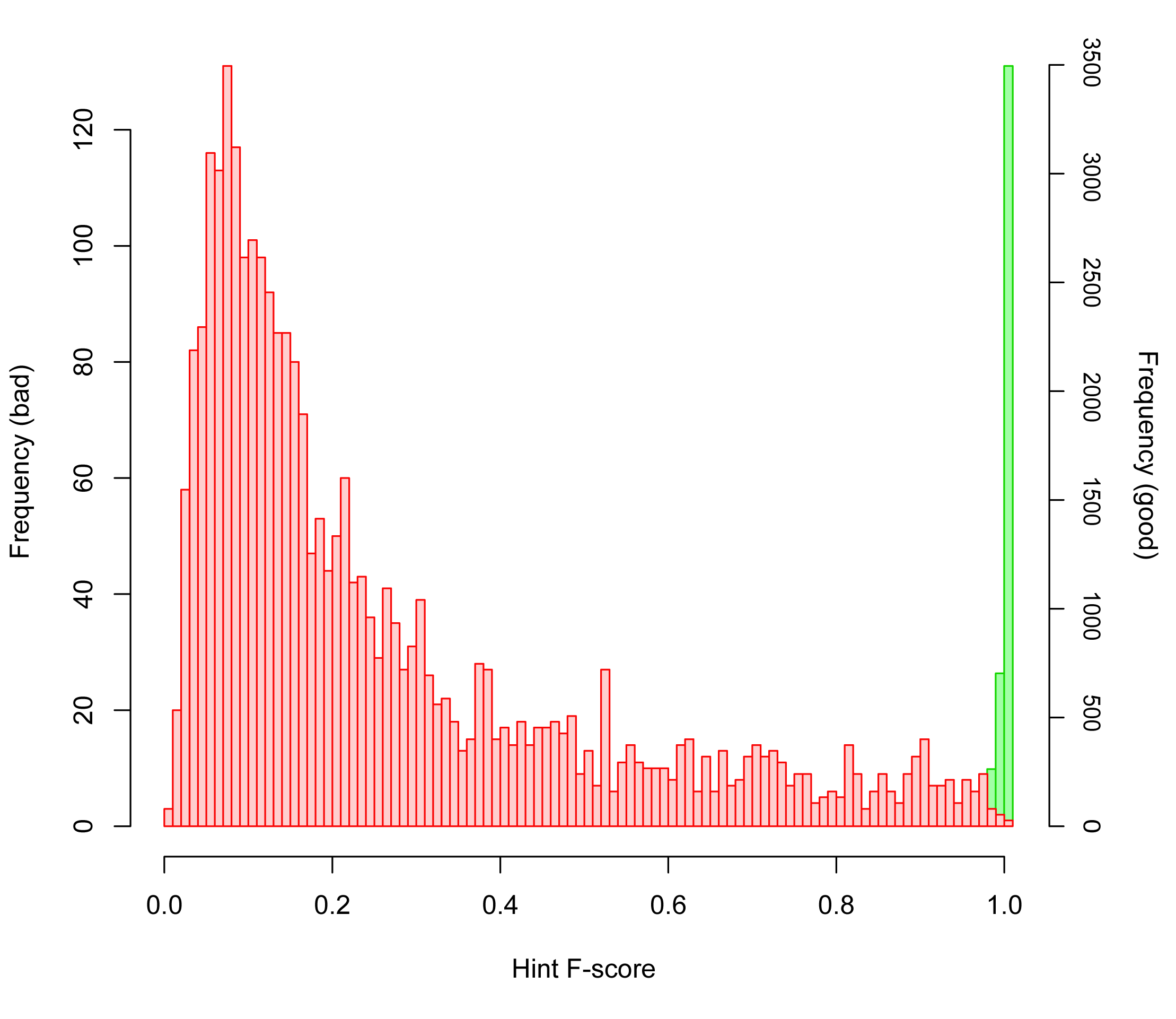

Supplement: Supplementary file 4 — Example distribution of hint F-scores for good vs. bad hints. Here, Augustus has been allowed to predict genes that are already present in the input genome, hence we can consider separately the good and bad hits as hints. Hits and hints are from running OrthoFiller on the five fungal species described in Table 1. Shown are the distributions of hint F-scores for good (green) and bad (red) hits respectively, demonstrating that practically all of the genuine hints have a hint F-score of 0.8 or higher. (TIF 1424 kb) [file 12864_2017_3771_MOESM4_ESM.tif]
